# Intra-coronary administration of tacrolimus markedly attenuates infarct size and preserves heart function in porcine myocardial infarction

**DOI:** 10.1186/1476-9255-9-21

**Published:** 2012-06-01

**Authors:** Sarah Chua, Steve Leu, Jiunn-Jye Sheu, Yu-Chun Lin, Li-Teh Chang, Ying-Hsien Kao, Chia-Hung Yen, Tzu-Hsien Tsai, Yung-Lung Chen, Hsueh-Wen Chang, Cheuk-Kwan Sun, Hon-Kan Yip

**Affiliations:** 1 Division of Cardiology, Department of Internal Medicine, Kaohsiung Chang Gung Memorial Hospital and Chang Gung University College of Medicine, Kaohsiung, Taiwan; 2 Center for Translational Research in Biomedical Sciences, Kaohsiung Chang Gung Memorial Hospital and Chang Gung University College of Medicine, Kaohsiung, Taiwan; 3 Division of Cardiovascular Surgery, Department of Surgery, Kaohsiung Chang Gung Memorial Hospital and Chang Gung University College of Medicine, Kaohsiung, Taiwan; 4 Department of Medical Research, E-Da Hospital, I-Shou University, Kaohsiung, Taiwan; 5 Basic Science, Nursing Department, Meiho University, Pingtung, Taiwan; 6 Department of Biological Science and Technology, National Pingtung University of Science and Technology, Pingtung, Taiwan; 7 Department of Biological Sciences, National Sun Yat-Sen University, Kaohsiung, Taiwan; 8 Department of Emergency Medicine, E-Da Hospital, I-Shou University, Kaohsiung, Taiwan

## Abstract

**Background:**

We test the hypothesis that intra-coronary tacrolimus administration can limit infarct size and preserve left ventricular ejection fraction (LVEF) after acute myocardial infarction (AMI) through ligating left anterior descending coronary artery (LAD) in mini-pigs.

**Methods:**

Twelve male mini-pigs were randomized into AMI-saline (MI-only) group and AMI-tacrolimus (MI-Tac) group that received intra-coronary saline (3.0 mL) and tacrolimus (0.5 mg in 2.5 mL saline) injection, respectively, beyond site of ligation 30 minutes after LAD occlusion.

**Results:**

Larger infarct area was noted in MI-only group (p < 0.001). Inflammatory biomarkers at protein [oxidative stress, tumor necrotic factor-α, nuclear factor-κB], gene (matrix metalloproteinase-9, plasminogen activator inhibitor-1), and cellular (CD40+, CD68+ inflammatory cells) levels were remarkably higher in MI-only animals (p < 0.01). Conversely, anti-inflammatory biomarkers at gene level (Interleukin-10), gene and protein level (endothelial nitric oxide synthase), and anti-oxidant biomarkers at both gene and protein levels [heme oxygenase 1, NAD(P)H:quinone oxidoreductase] were lower in MI-only group (p < 0.01). Number of apoptotic nuclei and apoptotic biomarkers expressions at gene and protein levels (Bax, caspase 3) were notably higher, whereas anti-apoptotic biomarkers at gene and protein levels (Bcl-2), LVEF, and fractional shortening were markedly lower in MI-only group (p < 0.001).

**Conclusion:**

Intra-coronary administration of tacrolimus significantly attenuated infarct size and preserved LV function.

## Background

Acute myocardial infarction (AMI) is the leading cause of death of patients hospitalized for cardiovascular disease [1,2]. Left ventricular (LV) remodeling as a consequence of LV chamber dilatation and pump failure following AMI, which are adverse factors typically associated with infarct size, largely accounts for unfavorable outcomes [3-5]. The cause of propagation of myocardium from injury to irrepressible death after AMI has been extensively investigated [6-8]. A body of evidence has suggested that a complicated network linking inflammatory reaction, immune response, production of reactive oxygen species (ROS), and cascade of complement activation may account for the phenomenon of “propagation of myocardium from injury to irrepressible death” after AMI [9-13].

Tacrolimus is chemically a macrolide. Activation of the T-cell receptor normally increases intracellular calcium, which acts via calmodulin to activate calcineurin. Calcineurin then dephosphorylates the transcription factor, i.e. nuclear factor of activated T-cells (NF-AT), which moves to the nucleus of the T-cell and increases the activity of genes coding for interleukin (IL)-2 and related cytokines. Tacrolimus prevents the dephosphorylation of NF-AT [14] through reducing the peptidyl-prolyl isomerase activity by binding to the immunophilin FKBP12 (i.e. FK506 binding protein) to create a new complex. This FKBP12-FK506 complex interacts with and inhibits calcineurin, thereby inhibiting both T-lymphocyte signal transduction and IL-2 transcription [15]. Although the action of tacrolimus is similar to that of cyclosporine, studies have shown that tacrolimus had significantly greater 6-year graft survival and a higher projected graft half-life than those receiving cyclosporin [16].

Clinical observational and experimental studies have previously shown that cyclosporine therapy effectively reduced LV infarction size and preserved LV function after AMI [17,18]. In view of the fact that immune and inflammatory reactions are one of the major contributors to death of cardiomyocytes after AMI [6-13,17] and that tacrolimus is a more potent immunosuppressant compared to cyclosporine, we propose that tacrolimus may limit the extent of myocardial infarction and improve LV function in the setting of AMI. To test the hypothesis, we administered tacrolimus through the coronary arterial system in an acute left anterior descending artery (LAD) occlusion-induced mini-pig AMI model and assessed the differences in LV infarction size, viability of myocardium, and LV function as compared with the animals without treatment.

## Methods

### Ethics

All animal experimental procedures were approved by the Institute of Animal Care and Use Committee at our institute and performed in accordance with the Guide for the Care and Use of Laboratory Animals (NIH publication No. 85–23, National Academy Press, Washington, DC, USA, revised 1996).

### Animal model of AMI and rationale of drug dosage

Each male mini-pig (Taitung Animal Propagation Station, Livestock Research Institute, Taiwan), weighing 16–18 kg, was anesthetized by intramuscular injection of ketamine (15 mg/kg) and maintained in anesthetized condition using an inhalation of 1.5% isoflurane for the whole procedure. After being shaved on the chest, the mini-pig was placed in supine position on a warming pad at 37 °C and then received endotracheal intubation with positive-pressure ventilation (180 mL/min) with room air using a ventilator support (Sn: Q422ZO, SIMS PneuPAC, Ltd.) during the procedure. Electrocardiogram (ECG) monitor and defibrillator were connected to the chest wall of each mini-pig. One ample of amiodarone (150 mg) was intravenously given to each animal before the AMI-induction procedure to prevent the occurrence of malignant arrhythmia.

Under sterile conditions, the heart was exposed through mid-thoracotomy. The pericardium was gently removed and the mid-portion of LAD was ligated with 5–0 prolene suture just distal to the first diagonal branch. Regional myocardial ischemia is confirmed by typical changes in waveform on ECG monitor and the observation of rapid discoloration of myocardium from pink to gray over the anterior surface of left ventricle, together with the rapid development of akinesia and dilatation of at-risk area. AMI was confirmed by complete ECG following the procedure.

Mid-LAD ligations were performed in 16 mini-pigs. Two mini-pigs in each group succumbed to either ventricular tachycardia or ventricular fibrillation even after defibrillation. The remaining 12 mini-pigs recruited for this study were then categorized into AMI plus normal saline treatment (MI-only) group (n = 6) and AMI plus tacrolimus therapy (MI-Tac) group (n = 6). For the purpose of comparison at molecular-cellular levels, normal cardiac tissues were obtained from a group of six mini-pigs without receiving any treatment that served as the normal controls (N_C_).

To determine the optimal drug dosage for maximal efficacy and acceptable safety, three different dosages of tacrolimus were adopted for pilot study, including: 1) 1.0 mg; 2) 0.5 mg; and 3) 0.25 mg. Each dosage was tried on two mini-pigs. The highest dosage induced fatal malignant ventricular tachyarrhythmia, whereas the second and third dosages were found to be safe. Since the infarct size was remarkably smaller in animals receiving the second dosage compared with the third one, the second dosage (i.e. 0.5 mg/kg) was utilized in the current study. By 30 minutes after LAD ligation, intra-coronary injections of physiological saline (3.0 mL) in MI-only group and tacrolimus (0.5 mg in 2.5 mL physiological saline) in MI-Tac group were given through the LAD beyond the point of ligation. The muscle and skin of the chest wall were then closed in layers. The animals were allowed to recover on the warming pad under close observation.

### Functional assessment using echocardiography

The echocardiographic study was performed using iE33 (Philips Medical System, Bothell, WA) with S5 transducers. The end-systolic dimension (EDS) and end-diastolic dimension (EDD) were measured at mitral valve and papillary levels of left ventricle. Recordings were stored for off-line two-dimensional image analysis using a computer software (Q-lab v6; Philips Medical System). For each mini-pig, three consecutive beats were measured and averaged for each variable. With the animals in a supine position, left ventricular internal dimensions [i.e. end-systolic diameter (ESD) and end-diastolic diameter (EDD)] were measured according to the American Society of Echocardiography leading-edge method using at least three consecutives cardiac cycles. The LV ejection fraction (LVEF) was calculated as: LVEF (%) = [(LVEDd^3^-LVEDs^3^)/LVEDd^3^] x 100. All measurements were performed by an animal cardiologist blind to treatment and non-treatment groups.

Intra-observer variability was evaluated in a series of six mini-pigs by one observer who examined the recordings from each animal in a consecutive way. Intra-observer variability for LVEF was 2.5 ± 2.1%. All measurements were performed by a cardiologist blinded to the treatment and non-treatment groups.

### Measurement of infarct area at basal, middle, and apical levels of left ventricle, and infarcted wall thickness at papillary muscle level

The heart was removed from each mini-pig following intravenous injection of an overdose of potassium chloride. Repeated flushing of the coronary artery with normal saline for washing out the red blood cells was performed immediately following heart removal. Three cross-sections (1 cm in thickness) from the basal, middle, and apical level, respectively, were used for infarct area (IA) analysis in IM-only and MI-Tac animals after being stained with 2% triphenyltetrazolium chloride (TTC). Briefly, all heart sections were placed on a tray with a scaled vertical bar to which a digital camera was attached. The sections were photographed from directly above at a fixed height. The images obtained were then analyzed using Image Tool 3 (IT3) image analysis software (University of Texas, Health Science Center, San Antonio, UTHSCSA; Image Tool for Windows, Version 3.0, USA). The calculated IA was expressed as arbitrary unit for comparison. The rest of the cardiac tissue was then cut into pieces for specific studies.

To determine the impact of tacrolimus therapy on thickness of the infarcted LV wall, three cross-sections of left ventricle at papillary muscle level were made for each animal and three measurements were recorded on the thickest regions for each section. The mean thickness was obtained for each animal. All measurements were performed by a technician blinded to the treatment and non-treatment groups.

### Reverse transcription PCR analysis

Reverse transcription polymerase chain reaction (RT-PCR) was conducted using LighCycler TaqMan Master (Roche, Germany) in a single capillary tube according to the manufacturer’s guidelines for individual component concentrations. Forward and reverse primers were each designed based on individual exons of the target gene sequence to avoid amplifying genomic DNA.

### Isolation of mitochondria

The myocardium from IA was excised and washed with buffer A (100 mM Tris–HCl, 70 mM sucrose, 10 mM EDTA and 210 mM mannitol, pH 7.4). Samples were minced finely in cold buffer A and then incubated for 10 minutes. All samples were homogenized in an additional 3 mL of buffer A using a motor-driven grinder. The homogenate was centrifuged twice at 700 g for 10 minutes at 4 °C. The supernatant was again centrifuged at 8,500 g for 15 minutes, and the pellets were then washed with buffer B (10 mM Tris–HCl, 70 mM sucrose, 1 mM EDTA, and 230 mM mannitol, pH 7.4). The mitochondria-rich pellets were then collected and stored at −70 °C.

### Western blot analysis

Equal amounts (50μg) of protein extracts were loaded and separated by SDS-PAGE using 12% acrylamide gradients. After electrophoresis, the separated proteins were transferred electrophoretically to a polyvinylidene difluoride (PVDF) membrane (Amersham Biosciences). Nonspecific sites were blocked by incubation of the membrane in blocking buffer [5% nonfat dry milk in T-TBS (TBS containing 0.05% Tween 20)] for overnight. The membranes were incubated with the indicated primary antibodies [Bcl-2 (1: 200, Abcam), Caspase 3 (1: 4000, Abcam), α-smooth muscle actin (SMA) (1: 1000, Sigma), Bax (1: 1000, Abcam), connexin (Cx)43 (1: 2000, Chemicon), matrix metalloproteinase (MMP)-9 (1: 5000, Abcam), tumor necrotic factor (TNF)-α (1: 1000, Cell Signaling), nuclear factor (NF)-κB (1: 600, Abcam), endothelial nitric oxide synthase (eNOS) (1: 1000, Abcam), NAD(P)H:quinone oxidoreductase (NQO1) (1: 1000, Abcam), heme oxygenase 1 (HO-1) (1: 1000, Abcam), Actin (1: 10000, Chemicon )] for 1 hr at room temperature. Horseradish peroxidase -conjugated anti-rabbit immunoglobulin IgG (1: 2000, Cell Signaling) was used as a second antibody for 1 hr at room temperature. The washing procedure was repeated eight times within 1 hr, and immunoreactive bands were visualized by enhanced chemiluminescence (ECL; Amersham Biosciences) and exposure to Biomax L film (Kodak). For purposes of quantitation, ECL signals were digitized using Labwork software (UVP).

### TUNEL assay for apoptotic nuclei in peri-infarct area

For each mini-pig, six sections (three longitudinal and three transverse sections of LV myocardium) were analyzed by an in situ Cell Death Detection Kit [TdT-FragEL^TM^ DNA Fragmentation (Calbiochem)] according to the manufacture's guidelines. The TUNEL-positive cells was examined in three randomly chosen high-power field (400x) and normalized to the total number of cells divided by 18.

### Immunohistochemical (IHC) staining for CD40+ and immunohistofluorescent (IHF) staining for CD68+ cells and troponin I

Paraffin sections (3 μm thick) were obtained from LV myocardium of each animal. For identification of CD40+ cells, the sections were initially incubated in 3% hydrogen peroxide for blocking the action of endogenous peroxidase, and then further processed using BEAT Blocker Kit (Zymed Company, #50-300) with immersion in solutions A and B for 30 minutes and 10 minutes, respectively, at room temperature. Polyclonal rabbit antibodies against CD40 (dilution 1/100; Spring Bioscience) were then used, followed by application of SuperPicTure^TM^ Polymer Detection Kit (Zymed) for 10 minutes at room temperature. Finally, the sections were counterstained with hematoxylin. IHF staining was performed using primary antibodies to recognize CD68+ cells (dilution 1/100; Abcam) or troponin I (dilution 1/100; Abcam), followed by the application of FITC-conjugated secondary antibodies (dilution 1/200; molecular probe). For negative control experiments, the primary antibodies were omitted.

### Oxidative stress reaction of LV myocardium

The Oxyblot Oxidized Protein Detection Kit was purchased from Chemicon (S7150). The oxyblot procedure was performed as previously described [19]. The DNPH derivatization was carried out on 6 μg of protein for 15 minutes according to the manufacturer’s instructions. One-dimensional electrophoresis was carried out on 12% SDS/polyacrylamide gel after DNPH derivatization. Proteins were transferred to nitrocellulose membranes which were then incubated in the primary antibody solution (anti-DNP 1: 150) for 2 h, followed by incubation in second antibody solution (1:300) for 1 h at room temperature. The washing procedure was repeated eight times within 40 minutes. Immunoreactive bands were visualized by enhanced chemiluminescence (ECL; Amersham Biosciences) which was then exposed to Biomax L film (Kodak). For quantification, ECL signals were digitized using Labwork software (UVP). For oxyblot protein analysis, a standard control was loaded on each gel.

### Histological study of fibrosis area

Masson's trichrome staining was used for studying fibrosis of LV myocardium. Three serial sections of LV myocardium were prepared at 4 μm thickness by Cryostat (Leica CM3050S). The integrated area (μm^2^) of fibrosis in the slides was calculated using Image Tool 3 (IT3) image analysis software (University of Texas, Health Science Center, San Antonio, UTHSCSA; Image Tool for Windows, Version 3.0, USA). Three selected sections were quantified for each animal. Three randomly selected HPFs (400x) were analyzed in each section. After determining the number of pixels in each fibrotic area per HPF, the numbers of pixels obtained from the three HPFs were summed. The procedure was repeated in two other slides for each animal. The mean pixel number per HPF for each animal was then determined by summating all pixel numbers and dividing by 9. The mean the integrated area (μm^2^) of fibrosis in LV myocardium per HPF was obtained using a conversion factor of 19.24 (1 μm^2^ represented 19.24 pixels).

### Statistical analysis

Data are expressed as mean values ± SD or percentage (%). The significance of group differences was evaluated with t-test. Continuous variables among 3 groups were compared using one-way ANOVA followed by Tukey’s multiple comparison procedure. Statistical analysis was performed using SAS statistical software for Windows version 8.2 (SAS institute, Cary, NC). A probability value < 0.05 was considered statistically significant.

## Results

### Transthoracic echocardiographic findings

Table 1 shows the echocardiographic findings prior to the procedure and at day 14 after AMI induction. By day 0 prior to AMI induction, at both mitral valve and papillary levels, the left ventricular end-diastolic dimension (LVEDd), left ventricular end-systolic dimension (LVEDs), left ventricular end-diastolic volume (LVEVd), left ventricular end-systolic volume (LVEVs), left ventricular fractional shortening (LVFS) and left ventricular ejection fraction (LVEF) did not differ between animals without treatment (MI-Only) and those with tacrolimus treatment (MI-Tac). Additionally, these parameters at mitral valve level were also similar between these two groups of animals at day 14 after AMI induction. Furthermore, no difference was noted in LVEDs and LVEVs at papillary level between these two groups of animals at day 14 after AMI induction. However, at papillary level, LVEDd and LVEVd were significantly higher, whereas LVFS and LVEF were significantly lower in MI-only group than in MI-Tac group at day 14 after AMI induction. These findings suggest that tacrolimus treatment attenuated LV remodeling and preserved LV function following AMI.

**Table 1:** Transthoracic echocardiographic results

**Variables**	**Tacrolimus (+)**	**Tacrolimus (−)**	**p-value**
Mitral valve level (prior to AMI)			
LVEDd (mm)	34.6 ±1.46	35.2 ± 2.32	0.241
LVEDs (mm)	23.4 ±2.32	24.5 ± 1.85	0.457
LVEVd (mL)	50.6 ± 5.3	52.2 ± 5.8	0.316
LVEVs (mL)	16.1 ± 1.4	15.4 ± 1.2	0.459
LVFS (%)	33.6 ± 4.15	35.4 ± 4.63	0.583
LVEF (%)	68.1 ± 1.2	68.1 ± 3.8	0.987
Papillary muscle level (prior to AMI)			
LVEDd (mm)	33.4 ± 0.68	34.1 ± 0.75	0.419
LVEDs (mm)	19.4 ± 0.51	19.9 ± 0.59	0.781
LVEVd (mL)	44.0 ± 2.1	46.6 ± 2.3	0.263
LVEVs (mL)	12.0 ± 0.23	12.6 ± 0.54	0.675
LVFS (%)	32.0 ± 2.12	34.1 ± 1.06	0.358
LVEF (%)	72.7 ± 1.3	72.8 ± 1.9	0.885
Mitral valve level (at day 14 after AMI)			
LVEDd (mm)	35.7 ± 0.66	36.1 ± 0.91	0.472
LVEDs (mm)	22.3 ± 0.54	23.0 ± 0.94	0.424
LVEVd (mL)	53.2 ± 5.6	55.01 ± 4.1	0.649
LVEVs (mL)	16.0 ± 1.4	18.0 ± 1.2	0.105
LVFS (%)	38.2 ± 3.96	37.0 ± 4.24	0.312
LVEF (%)	68.1 ± 1.2	67.2 ± 1.4	0.645
Papillary muscle level (at day 14 after AMI)			
LVEDd (mm)	36.5 ±1.48	37.9 ± 1.67	0.217
LVEDs (mm)	26.2 ± 3.03	31.4 ± 2.76	0.021
LVEVd (mL)	56.6 ±5.8	61.4 ± 6.8	0.263
LVEVs (mL)	26.2 ±8.9	39.6 ± 8.1	0.038
LVFS (%)	30.4 ± 5.94	21.8 ± 6.46	0.046
LVEF (%)	54.2 ±12.6	35.7 ±10.3	0.034

Data are expressed as means ± SD.

(+): with; (−): without.

AMI = acute myocardial infarction; LVEDd = left ventricular end-diastolic dimension; LVESd = left ventricular end-systolic dimension; LVEVd = left ventricular end-diastolic volume; LVEVs = left ventricular end-systolic volume; LVFS = left ventricular fractional shortening; LVEF = left ventricular ejection fraction.

### Measurement of infarct area at basal, middle, and apical levels of left ventricle, the wall thickness at papillary level of left ventricle and pathological findings using H. & E. stain

**Figure 1:**
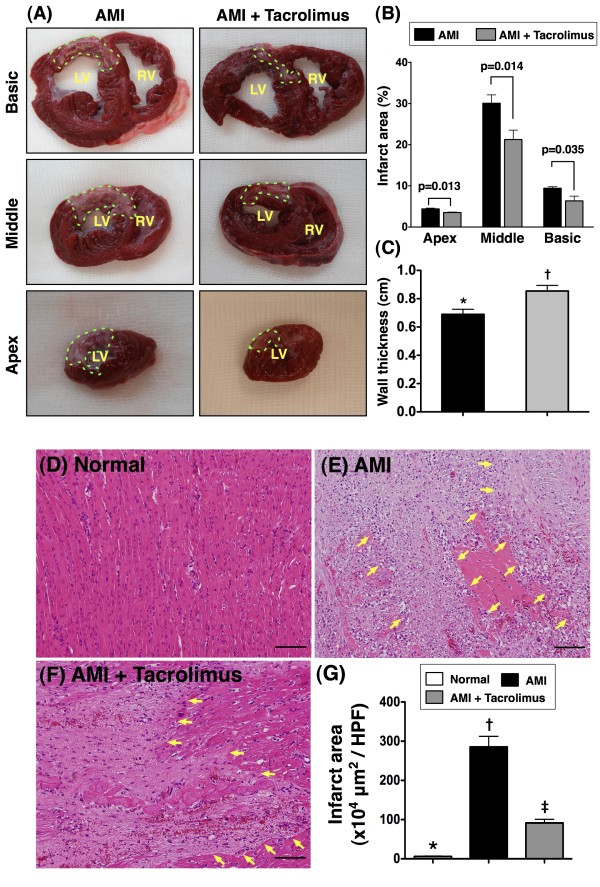
**Morphological and histopathological changes in infarcted area.Upper panel)** Identification of infarct area (IA) and infarct wall thickness at papillary level of left ventricle by TTC stain (n = 6 in each group). **A)** Triphenyltetrazolium chloride (TTC) (2.0%) staining for identifying IA (green dotted line). **B)** Significantly larger IA at basal, middle, and apical levels in acute myocardial infarction (AMI) plus normal saline group than in AMI plus tacrolimus therapy group. **C)** The wall thickness was significantly higher in AMI + tacrolimus than AMI group. * vs. †, p = 0.036. **Lower panel)** The results of H. & E. stain (100x) of the IA at middle level of left ventricle by day 14 after AMI induction (n = 6). **D to F)** H. & E. stain showing the IA (yellow arrows) was remarkably larger in MI-only than in MI-Tac group. **G)** * vs. other groups, P < 0.0001. All statistical analyses using one-way ANOVA, followed by Tukey’s multiple comparison procedure. Symbols (*, †, ‡) indicate significance (at 0.05 level). The Scale bars in right lower corner represent 100 μm. HPF, high-power field.

Upper Panel of Figure 1 shows the results of TTC staining by day 14 following AMI. As shown on the left panel (Figure 1A), IA was notably larger in MI-only than in MI-Tac group at basal, middle, and apical levels. Accordingly, quantification of the IA (Figure 1B) demonstrated significantly larger infarct at all three levels in the former. Additionally, the wall thickness at papillary level was significantly reduced in MI-only than in MI-Tac (Figure 1C).

The lower panel of Figure 1 shows the results of H. & E. stain of the IA at middle level of left ventricle by day 14 after AMI induction. As demonstrated on Figure 1D, 1E and 1F, the IA was remarkably larger in MI-only than in MI-Tac group. Accordingly, quantification of the IA (Figure 1G) revealed significantly larger infarct at middle level of left ventricle in the former.

These findings imply that, as compared with AMI without treatment, therapy using tacrolimus markedly limited infarct size and preserved infarcted wall thickness of the animals on day 14 after AMI.

### Quantification of viable myocardium and fibrosis in infarct area of LV myocardium

**Figure 2:**
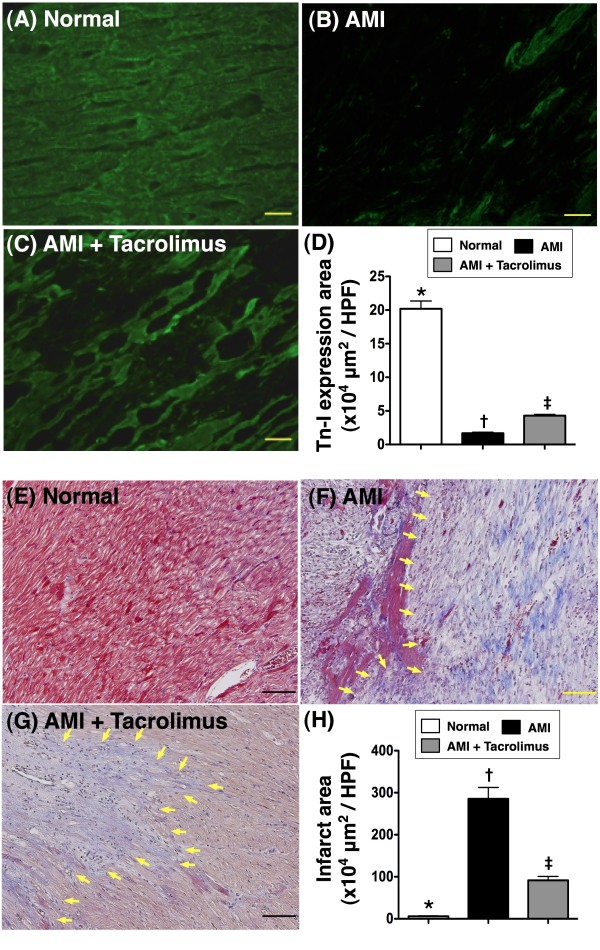
**Quantification of viable myocardium and fibrosis of myocardium in left ventricular infarct area. A to C)** Showing immunofluorescence (400x) troponin-I positive staining of IA for detecting viable myocardium, demonstrating markedly decreased distribution of troponin-I positively stained myocardium in IA of AMI group than that in AMI + tacrolimus group on day 14 following AMI. **D)** * vs. other groups, p < 0.0001. (n = 6 for each group) **E to G)** The mean fibrotic area in IA was remarkably higher in AMI group than in AMI + tacrolimus group on Masson Trichrome staining (blue color in IA) (400x). This finding suggests that tacrolimus therapy effectively inhibited fibrosis in IA (yellow arrows) after AMI. **H)** * vs. other groups, p < 0.0001. All statistical analyses using one-way ANOVA, followed by Tukey’s multiple comparison procedure. Symbols (*, †, ‡) indicate significance (at 0.05 level) (n = 6 for each group). The Scale bars in right lower corner represent 20 μm. HPF, high-power field.

Figure 2A, 2B and 2C indicates troponin-I staining of normal and IA for detecting viable myocardium, respectively. The immunofluorescent imaging study demonstrated that the distribution of troponin-I positive myocardium in IA was markedly decreased in MI-only group compared with that in the MI-Tac group on day 14 following AMI (Figure 2D).

To determine the impact of tacrolimus therapy on fibrosis in IA, Masson Trichrome staining was performed (Figure 2E-G). As expected, the mean fibrotic area in IA was remarkably higher in MI-only than in MI-Tac animals on day 14 after AMI induction (Figure 2H).

### The mRNA and protein expressions of inflammatory and endothelial dysfunction markers, oxidative stress and anti-inflammatory biomarkers

**Figure 3:**
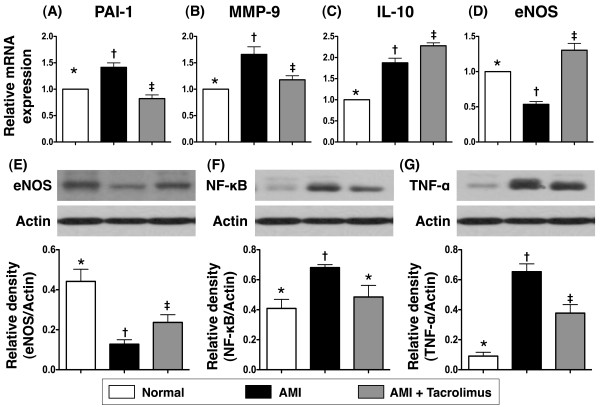
**Gene and protein expressions of inflammatory and anti-inflammatory biomarkers on day 14 after AMI in infarcted area. A & B)** Notably higher mRNA expressions of plasminogen activator inhibitor (PAI)-1 and matrix metalloproteinase (MMP)-9 in AMI than in normal and AMI + tacrolimus, and significantly higher in AMI + tacrolimus than in normal. * vs. other groups, p < 0.001. **C)** Significantly higher mRNA expression of interleukin (IL)-10 in AMI + tacrolimus than in AMI and normal, and remarkably higher in AMI than in normal. * vs. other groups, p < 0.0001. **D)** Remarkably higher mRNA expression of endothelial nitric oxide synthase (eNOS) in AMI + tacrolimus than in normal and AMI, and markedly increased in normal than in AMI. * vs. other groups, p < 0.001. **E)** Substantially higher protein expression of eNOS in normal than in AMI + tacrolimus and AMI, and notably higher in AMI + tacrolimus than in AMI. * vs. other groups, p < 0.01. **F)** Significantly higher protein expression of nuclear factor (NF)-κB (p65) in AMI than in normal and AMI + tacrolimus. * vs. other groups, p < 0.01. **G)** Significantly higher protein expression of tumor necrotic factor (TNF)-α in AMI than in normal and AMI + tacrolimus, and notably higher in AMI + tacrolimus than in normal. * vs. other groups, p < 0.001. All statistical analyses using one-way ANOVA, followed by Tukey’s multiple comparison procedure. Symbols (*, †, ‡) indicate significance (at 0.05 level).

The mRNA expressions of plasminogen activator inhibitor (PAI)-1 (Figure 3A) and matrix metalloproteinase (MMP)-9 (Figure 3B), two indexes of inflammation, were remarkably higher in MI-only compared to normal control animals (N_C_) on day 14 after AMI. However, these two markers were significantly lower in the MI-Tac group than in MI-only animals. These findings suggest that tacrolimus plays an essential role in suppressing the inflammatory reaction after AMI.

Interestingly, the mRNA expression of interleukin (IL)-10 (Figure 3C), an anti-inflammatory marker, was notably higher in MI-only than in N_C_ animals at day 14 after AMI induction. This biomarker was further increased in MI-Tac group compared with that in the MI-only group. Possible explanation may be a triggering of inherent self-protection in response to myocardial injury that was further enhanced by the suppression of immune/inflammatory reactions through tacrolimus therapy.

**Figure 4:**
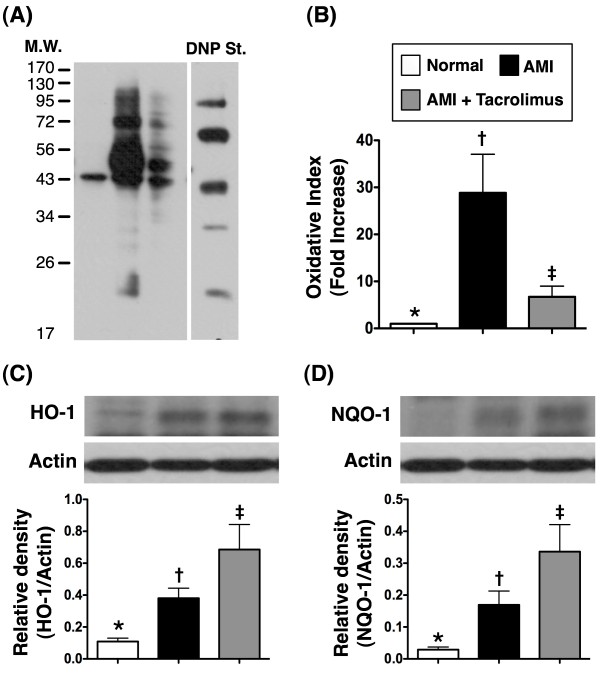
**Expression levels of oxidative index and anti-oxidant protein in infarcted area. A & B)** The protein expression of oxidative stress (n = 6). Oxidative index, protein carbonyls, was substantially higher in AMI than in normal and AMI + tacrolimus, and remarkably higher in AMI + tacrolimus than in normal. * vs. other groups, p < 0.0001. **C & D)** Expression levels of anti-oxidant proteins (n = 6). Protein expression of heme oxygenase (HO)-1 **(C)** and NAD(P)H:quinone oxidoreductase (NQO)-1 **(D)** were notably higher in AMI + tacrolimus than in AMI and normal, and significantly higher in AMI than in normal. * vs. other groups, p < 0.005. All statistical analyses using one-way ANOVA, followed by Tukey’s multiple comparison procedure. Symbols (*, †, ‡) indicate significance (at 0.05 level).

The gene and protein expressions of endothelial nitric oxide synthase (eNOS) (Figure 3D and E), an indicator of the integrity of endothelial function/vasodilatation as well as an anti-inflammatory contributor, were remarkably reduced in MI-only animals than in N_C_, but they were substantially increased in MI-Tac animals compared to their MI-only counterparts. Besides, the protein expressions of NF-κB (Figure 3F) and TNF-α (Figure 3G), two indicators of inflammation, were significantly increased in MI-only group than in N_C_, but they were markedly reduced in MI-Tac group as compared with MI-only animals. Furthermore, oxidative stress (Figure 4A and B) was significantly increased in MI-only group than in N_C_, but it was markedly reduced in MI-Tac than in MI-only animals by day 14 after AMI. Moreover, HO-1 (Figure 4C) and NQO1 (Figure 4D), two anti-oxidative biomarkers, were remarkably higher in MI-Tac animals than in MI-only and N_C_ animals, and significantly higher in MI-only group than in N_C_ animals on day 14 after AMI induction. These findings further support that AMI-elicited vigorous inflammatory reaction and oxidative stress, which cause further myocardial damage and deterioration of heart function, were effectively suppressed by tacrolimus.

### The mRNA and protein expressions of apoptotic and anti-apoptotic biomarkers

**Figure 5:**
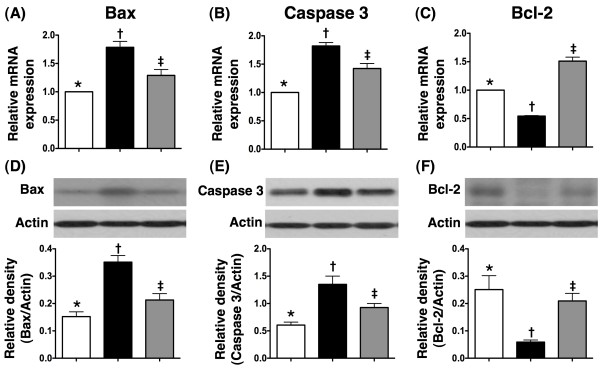
**mRNA and protein expressions of apoptosis and anti-apoptosis factors in infarcted area.** The mRNA and protein expressions of Bax (**A & D**) and caspase 3 (**B & E**) were notably higher in AMI than in normal and AMI + tacrolimus, and significantly higher in AMI + tacrolimus than in normal. Conversely, the mRNA expression of Bcl-2 (**C**) was remarkably lower in AMI than in normal and AMI + tacrolimus, and notably lower normal than in AMI + tacrolimus. Besides, the protein expression of Bcl-2 (**F**) was notably lower in AMI than in normal and AMI + tacrolimus, and significantly lower in AMI + tacrolimus than in normal. * vs. other groups, p < 0.001. All statistical analyses using one-way ANOVA, followed by Tukey’s multiple comparison procedure. Symbols (*, †, ‡) indicate significance (at 0.05 level) (n = 6 for each group).

**Figure 6:**
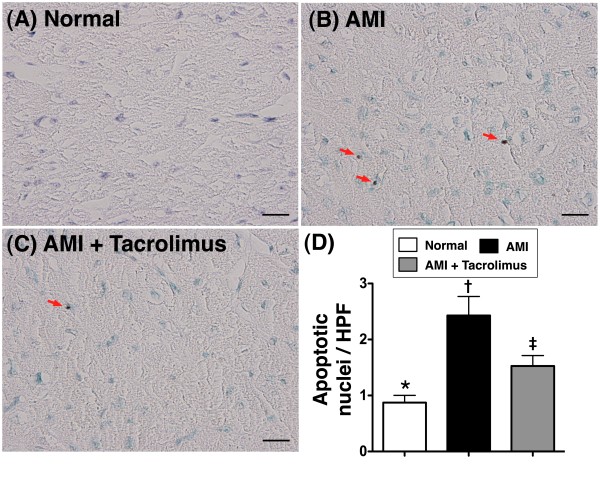
**Detection of apoptotic nuclei in peri-infarcted area. A to C)** showing the TUNEL examination of apoptotic nuclei (red arrows) in peri-infarct area on day 14 after AMI. **D)** The number of apoptotic nuclei was notably higher in AMI than in normal and AMI + tacrolimus, and significantly higher in AMI + tacrolimus than in normal. * vs. other groups, p < 0.001. All statistical analyses using one-way ANOVA, followed by Tukey’s multiple comparison procedure. Symbols (*, †, ‡) indicate significance (at 0.05 level) (n = 6 for each group). Scale bars in right lower corner represent 20 μm. HPF, high-power field (400x).

Both mRNA and protein expressions of Bax (Figure 5A and D) and caspase 3 (Figure 5B and E), two indicators of apoptosis, were notably higher in MI-only group than in N_C_, but were much lower in MI-Tac than in MI-only animals at day 14 after AMI induction. Conversely, both mRNA and protein expressions of Bcl-2 (Figure 5C and F), an index of anti-apoptosis, were remarkably lower in MI-only group than in N_C_, but were significantly higher in MI-Tac as compared with MI-only animals. Besides, TUNEL assay (Figure 6) showed substantially higher number of apoptotic nuclei in peri-IA in MI-only group than in N_C_, but notably lower number in MI-Tac compared with MI-only animals. These findings imply that tacrolimus suppressed myocardial damage through inhibiting cellular apoptosis after AMI.

### The protein expressions of connexin43 (Cx43) and α-smooth muscle actin (SMA) in IA

**Figure 7:**
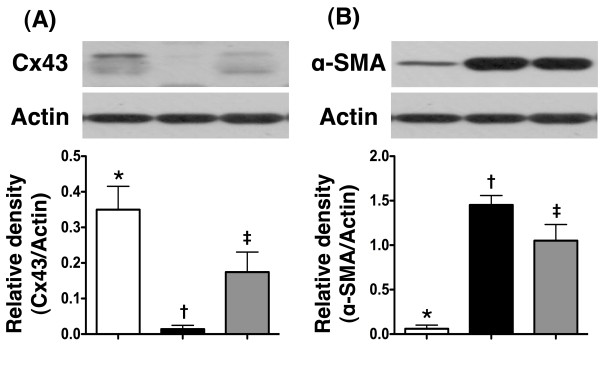
**Protein expressions of connexin43 and α-smooth muscle actin in infarcted area. A)** Showing protein expression of connexin43 (Cx43) remarkably lower in AMI than in normal and AMI + tacrolimus, and significantly lower in AMI + tacrolimus than in normal (n = 6), * vs. other groups, p < 0.001. **B)** Demonstrating protein expression of α-smooth muscle actin (SMA) remarkably higher in AMI than in normal and AMI + tacrolimus, and markedly increased in AMI + tacrolimus than in normal, * vs. other groups, p < 0.0001. All statistical analyses using one-way ANOVA, followed by Tukey’s multiple comparison procedure. Symbols (*, †, ‡) indicate significance (at 0.05 level) (n = 6 for each group).

IHC staining demonstrated that the protein expression of Cx43 (Figure 7A), a component of gap junction for providing pathways with minimal resistance for inter-cellular electrical coupling, was markedly reduced in MI-only group than in N_C_, but was notably up-regulated in MI-Tac compared with MI-only animals by day 14 after AMI. In contrast to Cx43 expression, the protein expression of α-SMA (Figure 7B), indicator of cardiac fibroblast proliferation in response to AMI, was substantially increased in MI-only animals than in N_C_, but it was significantly attenuated in MI-Tac than in MI-only group.

### Identification of inflammatory cells in IA

**Figure 8:**
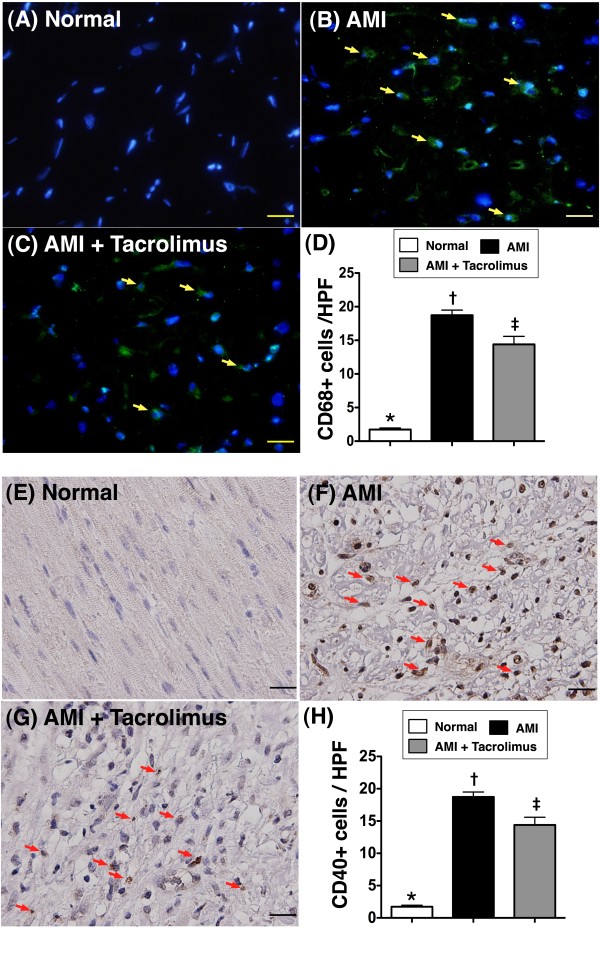
**Identification of inflammatory cells in infarcted area.** Immunofluorescence stain (**A to C**) showing the number of CD68+ stained cells (yellow arrows), an indicator of macrophage, markedly increased in AMI than in normal and AMI + tacrolimus, and significantly higher in AMI + tacrolimus than in normal. * vs. other groups, p < 0.0001 (**D**). Blue color indicated DAPI stain for the nuclei. Immunohistochemical stain (**E to G**) showing the number of CD40+ stained cells (red arrows), an index of inflammatory cells, substantially higher in AMI than in normal and AMI + tacrolimus, and notably higher in AMI + tacrolimus than in normal. * vs. other groups, p < 0.0001 (**H**). All statistical analyses using one-way ANOVA, followed by Tukey’s multiple comparison procedure. Symbols (*, †, ‡) indicate significance (at 0.05 level) (n = 6 for each group). HPF = high-power field (400x). The Scale bars in right lower corner represent 20 μm.

To evaluate whether CD40+ cells (Figure 8A-D) and CD68+ cells (Figure 8E-H) (macrophage surface marker), two surface markers of inflammatory cells, were up-regulated in the IA, IHC and IHF staining were performed, respectively. As anticipated, the numbers of CD40-positive and CD68-positive cells were substantially higher in MI-only group than in N_C_. However, they were found to be notably suppressed in MI-Tac animals than in those with MI only.

### Tentatively compared the effect of cyclosporine^18^ versus tacrolimus therapy on attenuating the inflammatory, apoptotic and oxidative-stress biomarkers in IA at day 14 after AMI

This study did not performed cyclosporine-treated AMI group. Thus, we utilized the results from our recent report [18] to compare the impact of cyclosporine and tacrolimus therapy on ameliorating the inflammatory, apoptotic and oxidative-stress responses after AMI. The results in Table 2 exhibited the superior effects of tacrolimus than that of cyclosporine on suppressing majority of these biomarkers.

**Table 2:** Comparison of the effect between cyclosporine and tacrolimus on attenuating expressions of inflammatory and oxidative-stress biomarkers in left ventricular myocardium at day 14 after acute myocardial infarction

**Variables**	**Cyclosporine (vs. Control)†**	**Tacrolimus (vs. Control)**
Caspase-3 mRNA	36% ↓	22%↓
Bax mRNA	11% ↓	24%↓↓*
Bcl-2 mRNA	75% ↑	200%↑↑*
MMP-9 mRNA	11%↓	29%↓↓*
eNOS mRNA	36%↑	160%↑↑*
No. of CD40+ cells	44%↓	22%↓
Oxidative stress index	45%↓	71%↓↓*

* indicated the more effective response in Tacrolimus therapy as compared with cyclosporine therapy on the changes of apoptotic, inflammatory and oxidative-stress biomarkers.

† Results from recent reported in the **Int J Cardiol** (2011;147:79–87) (reference no: 18).

MMP = matrix metalloproteinase; eNOS = endothelial nitric oxide synthase.

## Discussion

The present study, which investigated the therapeutic potential of tacrolimus via intra-coronary administration in a mini-pig AMI model, yielded several striking implications. First, tacrolimus therapy markedly attenuated myocardial infarct size. Second, remarkable inhibition of AMI-associated inflammatory responses was noted following tacrolimus administration. Third, cellular apoptotic episodes were substantially reduced by adopting this agent in the setting of AMI. Finally, AMI-induced LV remodeling was significantly limited and LV function was remarkably preserved utilizing this therapy.

Previous studies [9,10,18,20] have shown that the development of AMI quickly triggers inflammatory and immune responses, which, in turn, further elicit the complement cascade and enhance ROS generation. Histopathological studies [9,11-13,18] have demonstrated that inflammatory cells, including neutrophil, macrophages, T and B cells, are recruited and accumulate in IA where they secrete cytokines and produce antibodies specific to myosin and actin. Therefore, myocardial damage from AMI is often progressive and irreversible [11,13,18]. One important finding in the present study is the notable higher inflammatory reactions as reflected in the enhanced gene and protein expressions of pro-inflammatory cytokines (PAI-1, MMP-9, TNF-α and NF-κB), oxidized protein, IHC and IHF staining of inflammatory cells (CD40+ cells, CD68+ cell) in AMI animals than in normal controls. In this way, our findings reinforce those of the previous studies [9-11,13,18,20].

The principal finding in the current study is that, as compared to the animals with AMI without treatment, those received tacrolimus therapy had significantly lower levels of oxidative stress and inflammatory responses. These findings imply that tacrolimus possesses potent immunosuppressive property [15,16] against inflammatory reaction and ROS generation. More importantly, the results of present study, in addition to supporting the results of previous studies [9,11,13,18,20], could, at least in part, account for the reduction in infarct size, attenuation of LV remodeling, and preservation of LV function following tacrolimus administration.

Another important finding in the present study is that AMI-associated enhancement in apoptosis at both molecular and cellular levels, as demonstrated in the elevated pro-apoptotic gene and protein expressions (Bax, caspase 3) and increased number of apoptotic nuclei on TUNEL assay, was markedly reduced after tacrolimus treatment. Consistently, the suppressed gene and protein expressions of Bcl-2, an anti-apoptotic index, in AMI animals without treatment were notably preserved following tacrolimus treatment. These findings support that tacrolimus therapy, in addition to being anti-inflammatory, also effectively contributes to inhibition of AMI-related cellular apoptosis [18,20,21].

Pathologically, TTC staining demonstrated remarkably reduced infarct size at basal, middle, and apical levels as well as significantly increased LV thickness in animals with tacrolimus treatment compared to those without. Moreover, myocardial viability, as reflected in the positivity of troponin-I staining, was notably preserved in IA, whereas the fibrotic area on Masson Trichrome staining and cardiofibroblast proliferation (i.e. α-SMA) in IA were substantially reduced in animals having received tacrolimus. Furthermore, protein expression of Cx43 [19] was notably preserved in animals with tacrolimus treatment, suggesting preservation of electrical coupling between cardiomyocytes following AMI. Taking together, these findings may explain the significant attenuation of LV remodeling (i.e. LVEDd & LVEVd) and preservation of LV function (i.e. LVFS and LVEF) in animals with tacrolimus treatment.

Despite of the previous studies have emphasized that the tacrolimus is a more potent immunosuppressant compared to cyclosporine for preventing long-term graft failure [16], there has no available data to address which one of these two drugs is better for preserving myocardium from ischemia-related damage in a setting of AMI. We, therefore, compared the effects of these two drugs by using the information from our recent report [18] and the results of the current study on inhibiting the inflammatory, apoptotic and oxidative-stress responses after AMI. The results (Table 2) displayed the superior effects of tacrolimus to cyclosporine on suppressing the majority of these biomarkers. In this way, the results from the analyses were comparable with the reports from the previous study [16].

### Study limitations

This study has limitations. First, the therapeutic benefits of cyclosporine, a calcineurin inhibitor similar to tacrolimus, in limiting myocardial infarction, preserving LV function, and improving clinical outcome have been previously reported [17,18], however, since we did not include the positive control group of cyclosporine in the present study, whether the mechanisms of tacrolimus treatment involved in the protection of myocardium from ischemic injury are similar to those of cyclosporine therapy remains unclear. Further experimental investigation comparing the effects of these immunosuppressants on the progression of myocardial infarction, therefore, would strengthen the significance and interest of the present study. Second, the mechanisms of tacrolimus therapy on reducing the inflammation and cellular apoptosis were not explored in the current study. However, previous study has shown that tacrolimus therapy suppressed T cell activation through the inhibition of NF-AT activation which, in turn, attenuated inflammatory reaction [14,15].

## Conclusion

This experimental study demonstrated that AMI induction through LAD ligation in a mini-pig model initiated a cascade of inflammatory processes and generation of oxidative stress which, in turn, caused further myocardial injury. Intra-coronary administration of tacrolimus markedly attenuated inflammatory reaction, limited the infarct size, inhibited LV dilatation and remodeling, as well as preserved LV function in the setting of AMI without reperfusion therapy.

## Competing interests

The authors declare that they have no competing interests.

## Authors’ contributions

All authors have read and approved the final manuscript.

SC, SL, CKS, JJS, and HKY designed the experiment, drafted and performed animal experiments. LTC, THT, YLC, YHK, and CHY were responsible for the laboratory assay and troubleshooting. HWC, SL and HKY participated in refinement of experiment protocol and coordination and helped in drafting the manuscript.
